# New Effect of the Administration of Chloroform

**Published:** 1853-04

**Authors:** 


*New Effect of the Administration of Chloroform.*—Dr. Guisard presented the following note for publication:

My child, a boy of three years, attacked by a painful phymosis, could not, or rather would not, perform the acts of micturition and defecation, from an apprehension of the excessive pains which the muscular contractions necessary to the acts would create in the diseased part. The consequence was a retention of urine and fecal matters. The 8th of July last he was upon the point of being operated upon for his phymosis by my confrère and friend, M. Rigal. In order to relieve the little patient of the pain of the operation, we administered chloroform. Scarcely had my child become insensible, and fallen into a state of general resolution, when the urine escaped by a large rapid jet, and the fecal matters, diluted by severa enemata given in the morning, soon followed with no less impetuosity. The bladder and the rectum were completely emptied. Have we not discovered, exclaimed M. Rigal and myself, much astonished, a new and precious therapeutic resource in cases of spasmodic retention of either the urine or fecal matters?

The next day, twenty-four hours after, my little patient, still prevented by fear of the violent pains, had not urinated. Instead of having recourse, as previously, to the sound and the able hand of M. Rigal, in presence of a full bladder, typanism of the abdomen, burning heat of the skin, the complaints of the child, his resistence to micturition in spite of entreaties, promises and threats, I laid him upon his mother's lap and administered by force a small quantity of chloroform. Scarcely had his limbs and the whole body, yielding to the action of the anæsthetic agent, fallen into a state of resolution, when the child urinated, as upon the previous day, in a perfect manner. But what was remarkable, the child was not completely insensible: he had preserved his intelligence and consciousness of exterior life, which was manifested by his saying to us that it no longer hurt him to make water.—*Union Medicale.*

